# Optimising Human Papillomavirus Vaccination Strategies for Faster Cervical Cancer Elimination

**DOI:** 10.1111/apa.70756

**Published:** 2026-09-05

**Authors:** Joakim Dillner

**Affiliations:** ^1^ Department of Clinical Sciences, Intervention & Technologies, Center for Cervical Cancer Elimination Karolinska Institutet Stockholm Sweden

## Abstract

**Aim:**

Cervical cancer elimination is a global public health goal, with vaccination against Human Papillomavirus (HPV) and HPV screening being two major prevention pillars. This review focuses on how vaccinations can be used in order to reach the timepoint of cervical cancer elimination faster.

**Methods:**

The concepts in previously published reviews on ‘faster cervical cancer elimination’ are summarised and explained.

**Results:**

Major concepts explained are: HPV vaccines prevent not only cancer precursors, but also the HPV infection itself resulting in public health gains that exceed the individual direct vaccine protection because of herd immunity. The effective population coverages of vaccine‐induced immunity required to reach the critical extinction level for infection elimination are moderate, in particular when gender‐neutral vaccination is used. Major suggested enabling strategies include catch‐up vaccinations of adolescents, one‐dose HPV vaccination and use of HPV prevalences to monitor and optimise HPV vaccination programs. Finally, cervical screening with HPV testing is predicted to be easier if there are no new HPV infections.

**Conclusion:**

The major challenge for faster cervical cancer elimination is to design HPV vaccination strategies that achieve an effective population immunity against the HPV infection as fast as possible.

## Summary

- A variety of different Human Papillomavirus (HPV) vaccination strategies that affect the timepoint for cervical cancer elimination are possible.
- The critical outcome for faster elimination is controlling the HPV infection itself.
- Evaluating vaccination strategies based on their effect on reducing HPV prevalences and adapting HPV screening strategies for the post‐vaccination era will be essential.

**Abbreviations**
HPV: Human Papillomavirus
WHO: World Health Organization

Globally, there are > 600 000 incident cases of cervical cancer and almost 300 000 deaths every year [1], despite the fact that highly effective prophylactic HPV vaccines [2] and HPV screening tests [3] have been available for several decades. The World Health Organisation (WHO) has declared a global call to action to eliminate cervical cancer as a public health problem (defined as an incidence of < 4 per 100 000 women), with HPV vaccination and HPV screening as two of the pillars of the elimination strategy [4]. First‐generation vaccines (that are still in use in many Low and Middle Income Countries) targeted the two most oncogenic HPV types, HPV16 and 18, responsible for 77% of cervical cancer cases globally [5]. Most High Income Countries currently use a nonavalent vaccine that targets an additional five high oncogenic HPV types (HPV31, 33, 45, 52 and 58), together responsible for a further 17% of cervical cancer cases globally [5]. Faster cervical cancer elimination by concomitant HPV vaccination and HPV screening has been proposed [6] and further elaborated on regarding that rapid control of HPV infection is the most important bottleneck [7]. The concepts have been reviewed previously [8].

Although the HPV vaccines were originally evaluated regarding prevention of HPV‐induced disease such as cervical cancer precursors [9], it soon became clear that the vaccines also prevented the infection itself [2]. This results in an important shift in the aim of HPV vaccination programs, as vaccine‐induced immunity will break transmission chains, with indirect effects at the population level becoming increasingly important. A review of dynamic transmission models of HPV found that most models predicted that gender‐neutral HPV vaccination at an 80% effective vaccination coverage would result in the infection declining to below the critical extinction level [10]. Models typically rely on assumptions, but a model that was based on the real herd immunity effects seen in a community randomised trial found similar, albeit somewhat more optimistic results: extinction was predicted at 70% effective vaccination coverage (both genders) or at 90% coverage in girls‐only vaccination programs [11]. Assessing which age groups need to be reached with this vaccination coverage requires estimation of the reproductive rate of the infection in different birth cohorts, as extinction requires a reduction of the reproductive rate to levels below 1 (not enough new infections to maintain the infection in the population) [7]. The reproductive rate can be readily calculated from the incidence of the infection [7]. The reproductive rate is dependent on the sexual contact patterns in a population, which may differ greatly between different populations both in terms of the rate of contacts, age‐related contact rates, and assortative mixing patterns (subjects in core groups preferentially contacting other subjects in the core groups). However, many populations have a peak in the rate of sexual contacts during adolescence. For example, in Sweden the peak occurs at 17–18 years of age, with successively decreasing rates with increasing age [7]. Population immunity was predicted to occur when birth cohorts up to 30 years of age are immune [7]. The duration of infectivity is another major determinant of the reproductive rate. The duration of HPV detectability using highly sensitive PCR methods has been extensively studied [12]. An infection typically becomes undetectable within 1 year, with HPV16 having the longest duration of detectability and more low risk viruses having shorter durations of detectability [12]. It is not known what amount of virus is required for infectivity, but given the very short duration also when considering any detectable HPV, results from modelling studies have been fairly consistent [10]. The short duration of detectability implies that once population immunity is established, propagation of HPV infection in the population should cease shortly thereafter [7].

Randomised vaccination trials of HPV vaccination of adolescent and adult women have found high vaccine efficacy among women testing HPV negative at baseline (about 89%) [13], but no effect against HPV infections that are detectable at the timepoint of vaccination [13]. For maximum efficacy, it is therefore recommended to vaccinate at an age before sexual debut, when the children are as yet unexposed to HPV [14]. However, even highly sexually active populations are not exposed to all nine types of HPV that can be prevented by vaccines and may thus benefit from vaccination. Somewhat surprisingly, many countries use catch‐up HPV vaccination programmes without HPV testing at baseline even though it is known that the protection after adult HPV vaccination is restricted to HPV‐negative women.

Single‐dose HPV vaccination strategies are widely used globally and officially recommended by WHO for children and adolescents [14]. However, optimal HPV vaccine dosing schedules among adults are insufficiently studied. Whereas a large body of evidence supports the use of 1 dose HPV vaccination strategies among children and adolescents, there are only a few dose reduction studies among women above 20 years resulting in that WHO recommends 1 dose HPV vaccination up to 20 years of age, but 2‐dose vaccination with prolonged dosing interval (at least 6 months) above 20 years of age [14]. One‐dose vaccination schedules would greatly simplify prevention using campaign strategies, thereby contributing to faster cervical cancer elimination. Quantification of the degree of efficacy of one‐dose HPV vaccination strategies among adults would be essential for the design of faster campaigns.

An important consideration regarding HPV vaccination of adult and adolescent populations is how the immunity will affect the prospects for effective HPV screening. As HPV vaccinations are effective in preventing new infections, not for treating existing ones, the population‐level effect of HPV vaccinations on cervical cancer incidence is achieved only with a considerable delay. By contrast, screening has no time component at all (if all women have been screened and either found to be negative or to have had existing precursors found and treated, the full effect of the screening program on invasive cancer protection is instant) and improving the prospects for effective screening is therefore a key component in achieving a faster cervical cancer elimination [6]. The concept was described by a European group of experts in 2016 and proposes administering HPV vaccine concomitantly with HPV screening in order to prolong the protective effect of testing HPV negative by reducing the amount of new infections [6]. It is well established that even after a single negative HPV test, the cancer risk is very low also for prolonged periods of time (up to 18 years) [3]. However, in younger age groups with a high incidence of new HPV infections it may be necessary to first reduce the HPV incidence in order to make HPV screening more effective.

In 2021, we extended the faster concept by proposing that concomitant HPV screening and HPV vaccination among adult women should be targeted to age groups where the infection is spreading with a reproductive rate of > 1, which in Sweden extended up to the age of 30 [7]. The HPV vaccination policies in Sweden have seen three clear phases: (i) on‐demand vaccinations (HPV vaccines recommended and subsidised, but no active invitations and co‐payment from the women required), which had low population coverage and limited effect on HPV prevalences in the population [15]. (ii) school‐based vaccination, where vaccines are systematically offered to girls in the ages 11–12 when they attend schools, which initially had only 83% population coverage but is nowadays above 90%. In these birth cohorts, the most oncogenic HPV types (16 and 18) are almost eliminated [15] and (iii) organised catch‐up vaccinations originally offered to girls 13–18 years of age by personal invitations, but administered out of schools. These had moderate vaccination coverages (between 50% and 60%), which reduced the prevalences of HPV vaccine types but was not enough to stop the circulation [15]. The finding that HPV is disappearing from highly vaccinated birth cohorts is a universal finding that appears to have been the case for every country where the HPV prevalences in highly vaccinated birth cohorts have been measured [16, 17]. The Swedish nationwide trial of faster cervical cancer elimination targeted all eligible women in the country aged 23–31 years with concomitant HPV vaccination and HPV screening [18], with about 64% attendance. Simultaneously, population‐based HPV screening with HPV self‐sampling at home was rolled out for all age groups (up to age 79 for women who were not previously HPV tested) [19]. There was a sharp decline in cervical cancer incidences, which was seen for all age groups offered self‐sampling but was particularly prominent in the age groups offered concomitant HPV vaccination and HPV screening [19].

The major advantage of focusing HPV vaccination programs on reducing the HPV prevalences is the effect that reduced HPV prevalences have on the feasibility of effective cervical screening. HPV infections used to be very common among sexually active adolescents—if considering all 14 HPV types screened for in first generation HPV screening tests, it was not uncommon to report HPV prevalences of 30% or more among women below the age of 30 [20], giving the impression that HPV screening has low specificity and needs to have a second (‘triaging’) screening test after the HPV test and/or be used only in an age‐restricted fashion focusing on older women. However, there are only 8 HPV types that cause more than 1% of cervical cancers [5] and the remaining formally oncogenic HPV types in first generation HPV tests are quite common in the general population, resulting in unreasonably high numbers of women needed to screen to prevent a case of cervical cancer [20]. Today, many HPV tests allow separation of high oncogenicity HPV types from low oncogenicity HPV types (so‐called ‘extended genotyping’) and in the future there will no doubt be HPV tests that do not test for low oncogenicity HPV types at all [21]. Second, in highly vaccinated birth cohorts HPV prevalences have declined to the extent that the most oncogenic HPV types have become rare [15, 17], resulting in that there is no longer need for triaging tests when testing positive for highly oncogenic HPVs. An important consequence of this is that cervical screening can be greatly simplified by the use of self‐sampling for HPV. The lower resources required for self‐sampling strategies results in that whole populations can be reached using limited‐time campaign strategies, avoiding the slow implementation of screening programs that screen only a part of the population at fixed screening intervals (requiring a whole interval of several years until the whole population has been offered screening and in most settings several intervals until acceptable population coverages of screening have been reached). Further, self‐sampling strategies result in higher population coverages of screening. The attendance is particularly high if ‘dispense and collect’ strategies are used, but they are improved also when comparing mail‐out self‐sampling at home to previously used strategies with sampling for screening at physical visits [22]. Thus, although faster elimination of the critical risk factor for cervical cancer (HPV prevalences) is an essential step towards the long‐term and sustainable elimination of cervical cancer, the most important effect of reduced HPV prevalences is that they allow more feasible and effective HPV screening programs with a predicted instant effect against cervical cancer incidences.

## Author Contributions

**Joakim Dillner:** conceptualization, investigation, funding acquisition, writing – original draft, methodology, validation, visualization, writing – review and editing, formal analysis, project administration, data curation, resources.

## Funding

This work was supported by Vetenskapsrådet, 2025‐00252, 2021‐01228. Cancerfonden, 201199UsF02H, 201198PjF01H. Center for Innovative Medicine, 976207, 20190809. Swedish Government.

## Conflicts of Interest

The author declares no conflicts of interest.

## Data Availability

Data sharing not applicable to this article as no datasets were generated or analysed during the current study.

## References

[1] H. Sung , A. M. Filho , M. Laversanne , et al., “Global Cancer Statistics 2024: GLOBOCAN Estimates of Incidence and Mortality Worldwide for 34 Cancers in 186 Countries,” CA: A Cancer Journal for Clinicians 76, no. 4 (2026): e70090, 10.3322/caac.70090. PMC13343830 42417444

[2] J. T. Schiller , X. Castellsagué , and S. M. Garland , “A Review of Clinical Trials of Human Papillomavirus Prophylactic Vaccines,” Vaccine 30 (2012): F123–F138, 10.1016/j.vaccine.2012.04.108. PMC4636904 23199956

[3] J. C. Gage , M. Schiffman , H. A. Katki , et al., “Reassurance Against Future Risk of Precancer and Cancer Conferred by a Negative Human Papillomavirus Test,” Journal of the National Cancer Institute 106, no. 8 (2014): dju153, 10.1093/jnci/dju153. PMC4111283 25038467

[4] World Health Organization and WHO , Global Strategy to Accelerate the Elimination of Cervical Cancer as a Public Health Problem (World Health Organization, 2020).

[5] F. Wei , D. Georges , I. Man , I. Baussano , and G. M. Clifford , “Causal Attribution of Human Papillomavirus Genotypes to Invasive Cervical Cancer Worldwide: A Systematic Analysis of the Global Literature,” Lancet 404, no. 10451 (2024): 435–444. 10.1016/S0140-6736(24)01097-3 39097395

[6] F. X. Bosch , C. Robles , M. Díaz , et al., “HPV‐FASTER: Broadening the Scope for Prevention of HPV‐Related Cancer,” Nature Reviews Clinical Oncology 13, no. 2 (2016): 119–132, 10.1038/nrclinonc.2015.146. 26323382

[7] J. Dillner , K. M. Elfström , and I. Baussano , “Prospects for Accelerated Elimination of Cervical Cancer,” Preventive Medicine 153 (2021): 106827. 10.1016/j.ypmed.2021.106827 34599922

[8] F. R. Burdier , F. X. Bosch , D. E. Waheed , et al., “Accelerating HPV‐Related Cancer Elimination ‐ a Meeting Report,” BMC Proceedings 19, no. Suppl 19 (2025): 22, 10.1186/s12919-025-00337-1. PMC12395638 40877872

[9] FUTUREII Study group , “Quadrivalent Vaccine Against Human Papillomavirus to Prevent High‐Grade Cervical Lesions,” New England Journal of Medicine 356, no. 19 (2007): 1915–1927, 10.1056/NEJMOA061741. 17494925

[10] M. Brisson , É. Bénard , M. Drolet , et al., “Population‐Level Impact, Herd Immunity, and Elimination After Human Papillomavirus Vaccination: A Systematic Review and Meta‐Analysis of Predictions From Transmission‐Dynamic Models,” Lancet Public Health 1, no. 1 (2016): e8–e17, 10.1016/S2468-2667(16)30001-9. PMC6727207 29253379

[11] M. Lehtinen , I. Baussano , J. Paavonen , S. Vänskä , and J. Dillner , “Eradication of Human Papillomavirus and Elimination of HPV‐Related Diseases ‐ Scientific Basis for Global Public Health Policies,” Expert Review of Vaccines 18, no. 2 (2019): 153–160. 10.1080/14760584.2019.1568876 30657348

[12] M. Zhao , P. Kang , L. Zhu , et al., “Global Pattern of Persistent Human Papillomavirus Infection in Female Genital Tract: An Update System Review and Meta‐Analysis,” iScience 27, no. 10 (2024): 110991, 10.1016/j.isci.2024.110991. PMC11519437 39474077

[13] M. Arbyn , L. Xu , C. Simoens , and P. P. L. Martin‐Hirsch , “Prophylactic Vaccination Against Human Papillomaviruses to Prevent Cervical Cancer and Its Precursors,” Cochrane Database of Systematic Reviews 5, no. 5 (2018): CD009069, 10.1002/14651858.CD009069.PUB3. PMC6494566 29740819

[14] World Health Organisation , “Human Papillomavirus Vaccines: WHO Position Paper (2022 Update),” Weekly Epidemiological Record 97, no. 50 (2022): 645–672.

[15] P. Gray , J. Wang , S. Nordqvist Kleppe , K. M. Elfström , and J. Dillner , “Population‐Based Age‐Period‐Cohort Analysis of Declining Human Papillomavirus Prevalence,” Journal of Infectious Diseases 231, no. 4 (2025): e638–e649, 10.1093/infdis/jiaf032. PMC11998575 39841153

[16] M. Drolet , É. Bénard , N. Pérez , M. Brisson , and HPV Vaccination Impact Study Group , “Population‐Level Impact and Herd Effects Following the Introduction of Human Papillomavirus Vaccination Programmes: Updated Systematic Review and Meta‐Analysis,” Lancet 394, no. 10197 (2019): 497–509, 10.1016/S0140-6736(19)30298-3. PMC7316527 31255301

[17] M. H. Nonboe , G. M. Napolitano , J. B. Schroll , et al., “Human Papillomavirus Prevalence in First, Second and Third Cervical Cell Samples From Women HPV‐Vaccinated as Girls, Denmark, 2017 to 2024: Data From the Trial23 Cohort Study,” Eurosurveillance 30, no. 27 (2025): 2400820. 10.2807/1560-7917.ES.2025.30.27.2400820 PMC12262111 40642768

[18] L. S. Arroyo Mühr , A. Gini , E. Yilmaz , et al., “Concomitant Human Papillomavirus (HPV) Vaccination and Screening for Elimination of HPV and Cervical Cancer,” Nature Communications 15, no. 1 (2024): 3679, 10.1038/s41467-024-47909-x. PMC11063066 38693149

[19] H. Andersson and J. Dillner , “Population‐Based Cervical Screening With Human Papillomavirus Self‐Sampling at Home and Incidence of Cervical Cancer in Sweden,” Preventive Medicine 206 (2026): 108540, 10.1016/j.ypmed.2026.108540. 41692262

[20] J. Wang , K. M. Elfström , C. Lagheden , et al., “Impact of Cervical Screening by Human Papillomavirus Genotype: Population‐Based Estimations,” PLoS Medicine 20, no. 10 (2023): e1004304, 10.1371/journal.pmed.1004304. PMC10637721 37889928

[21] J. Dillner , “Human Papillomavirus Screening: Design and Targeting of Human Papillomavirus Genotypes,” Current Topics in Microbiology and Immunology 448 (2026): 81–92, 10.1007/978-3-032-17775-9_4. 41691586

[22] K. M. Elfström and J. Dillner , “Towards Faster, Better, and Cheaper Cervical Cancer Screening,” Lancet Public Health 11, no. 2 (2026): e72–e73, 10.1016/S2468-2667(26)00002-2. 41611364

